# Instability of 24-hour intraocular pressure fluctuation in healthy young subjects: a prospective, cross-sectional study

**DOI:** 10.1186/1471-2415-14-127

**Published:** 2014-11-04

**Authors:** Yoo Kyung Song, Chang-Kyu Lee, Jiwon Kim, Samin Hong, Chan Yun Kim, Gong Je Seong

**Affiliations:** Institute of Vision Research, Department of Ophthalmology, Yonsei University College of Medicine, 50 Yonsei-ro, Seodaemun-gu, Seoul, 120-752 Republic of Korea; Department of Ophthalmology, Maryknoll Medical Center, Busan, Republic of Korea

**Keywords:** Blood pressure, Fluctuation, Glaucoma, Intraocular pressure

## Abstract

**Background:**

Elevated intraocular pressure (IOP) is a major risk factor for the development and/or progression of glaucoma, and a large diurnal IOP fluctuation has been identified as an independent risk factor of glaucoma progression. However, most previous studies have not considered the repeatability of 24-hour IOP measurements. The aim of this study was to evaluate the instability of 24-hour IOP fluctuations in healthy young subjects.

**Methods:**

Ten healthy young volunteers participated in this prospective, cross-sectional study. Each subject underwent 24-hour IOP and systolic/diastolic blood pressure (SBP/DBP) assessments both in sitting and supine positions every 3 hours, once a week for 5 consecutive weeks. Mean ocular perfusion pressure (MOPP) was then calculated for both positions. The intraclass correlation coefficients (ICCs) of maximum, minimum, and fluctuation parameters were computed for IOP, SBP/DBP, and MOPP. Fluctuation was defined as the difference between maximum and minimum values during a day.

**Results:**

Among the serial measurements taken over a 24-hour rhythm, the maximum/minimum values of IOP, as well as BP, showed excellent agreement: regardless of position, all ICC values were over 0.800. Most of the BP fluctuation values also showed excellent agreement. IOP fluctuation, however, did not show excellent agreement; the ICC of sitting IOP fluctuation was just 0.212. MOPP fluctuation also showed poor agreement, especially in the sitting position (ICC, 0.003).

**Conclusion:**

On a day to day basis, 24-hour IOP fluctuations were not highly reproducible in healthy young volunteers. Our results imply that a single 24-hour IOP assessment may not be a sufficient or suitable way to characterize circadian IOP fluctuations for individual subjects.

## Background

Elevated intraocular pressure (IOP) is a major risk factor for development and/or progression of glaucoma, and IOP reduction is a well-known treatment strategy for slowing the progression of the disease. However, due to the fact that IOP is not a constant value and it is affected by many internal and environmental factors, many glaucoma researchers have conducted studies to characterize its circadian rhythm and short/long-term variations [1–12].

Nevertheless, controversy exists as to whether IOP fluctuations are an independent predictive risk factor for the progression of glaucoma. In previous studies, large fluctuations in diurnal IOP were deemed independent risk factors for the progression of glaucoma [8, 13], while in other studies, diurnal fluctuations in IOP itself were not [6, 14]. Regardless of whether IOP fluctuations may or may not be a predictive or independent factor, the importance of understanding circadian IOP profiles in glaucoma patients is consensually agreed upon. However, in nearly all studies characterizing diurnal or circadian IOP patterns, there is little or no data to describe the repeatability of IOP patterns over time. In fact, Realini et al. [15, 16] only recently reported that both healthy subjects and glaucoma patients failed to demonstrate a repeatable diurnal IOP pattern on a daily basis. However, they only checked IOP during their office hours (from 08:00 AM to 08:00 PM) for just two days at one week apart.

In the present study, to better understand the instability of 24-hour IOP patterns, 24-hour IOP was measured for each participant once a week for 5 consecutive weeks. Due to the fact that IOP is known to be associated with blood pressure (BP) [17, 18], hemodynamic instability was also concurrently monitored. Additionally, to determine IOP patterns in the participants’ actual daily lives, patients were instructed to continue their lives as normal during the study period. They were not hospitalized and their sleep cycles were not controlled. The participants also were not prohibited from consuming caffeine or alcohol.

## Methods

### Participants

After obtaining approval of the Institutional Review Board of Gangnam Severance Hospital, Yonsei University College of Medicine, we recruited 10 healthy young female volunteers who were training as residents in various departments in our institute. The study was conducted in accordance with the tenets of the Declaration of Helsinki. All participants provided written informed consent to be enrolled in the study. Each subject received a comprehensive ophthalmic examination and interview, and no participant demonstrated any signs of ophthalmic and/or systemic diseases or had a family history of glaucoma.

### Measurements

At first, IOP was measured in a sitting position using Goldmann applanation tonometry (GAT) and a Tono-Pen AVIA tonometer (Reichert Technologies Inc., Depew, NY, USA) after the subject had been seated for at least for 5 minutes. IOP was then measured in a supine position using the Tono-Pen AVIA tonometer after the subject had remained in position for at least another 5 minutes. Before each IOP measurement, a drop of 0.5% proparacaine hydrochloride ophthalmic solution was inserted into the eyes as a local anesthetic. To minimize the effect of a possible transient lowering of IOP following applanation tonometry, we took readings at an interval of 5 minutes. A single clinician measured the IOPs. To avoid bias, previous IOP values were completely masked to the clinician and the statistical analyses were performed by an independent person. The IOP data obtained only from the right eye of each subject was finally analyzed.

Systolic and diastolic BPs (SBP and DBP, respectively) were measured on the upper left arm by an automated oscillometric device after the subjects had been seated for at least 5 minutes and had been lying for at least 5 minutes, correspondingly. All the subjects were evaluated by the same person using the same technique from visit to visit. Subjects were allowed to continue with their normal activities and to consume normal amounts of food and fluids, including caffeine and alcohol. Their daily lives including sleep were not controlled or influenced in anyway.

All participants were measured 1 week apart for 5 weeks when they were on duty as residents in training over a 24 period in our institute. Measurements of IOP and BPs were taken every 3 hours over a 24 hour period, once a week for 5 consecutive weeks. For each measurement, IOP and BPs were checked three times a day, and the average of the three values was recorded. Mean ocular perfusion pressure (MOPP) was also calculated as follows: MOPP = 2/3× [DBP + {1/3 × (SBP - DBP)}] - IOP [19]. For each day, three parameters of maximum, minimum, and fluctuations were determined for IOP, BPs, and MOPP in both the sitting and supine positions. Fluctuation was defined as the difference between the maximum and minimum measurements.

### Statistical analysis

Student's paired t-test, Pearson's coefficient, and linear regression coefficient of determination were used to compare and correlate lOP measurements between GAT and Tono-Pen AVIA tonometer. Agreement between the two tonometers was also calculated according to the difference between appropriate pairs of values for each subject against the mean of the two measures by Altman-Bland's method. To assess the reproducibility of IOP, BPs, and MOPP, intraclass correlation coefficients (ICCs) were calculated for each of their maximum, minimum, and fluctuation values. The ICCs were computed as the ratio of the between-subject component of variance to the total variance, and indicated as the proportion of variance in the measurements due to differences among the subjects. ICC values near 1.00 reflect little variation in the measurements obtained for the same subject, compared with measurements obtained for different subjects. ICC values less than 0.40 represent poor agreement beyond chance, whereas ICC values from 0.40 to 0.75 signify fair to good agreement beyond chance and ICC values greater than 0.75 indicate excellent agreement beyond chance [20]. Negative ICC values reveal greater within-subject variability than between-subject variability, representing agreement that is even less than expected by chance alone. The Statistical Package for Social Sciences (SPSS) version 18.0 (SPSS Inc., Chicago, IL, USA) was used for all statistical analysis. All *P*-values less than 0.05 were considered statistically significant.

## Results

Overall, 10 healthy female Korean volunteers (mean age; 27.25 ± 1.75 years old) were enrolled in this study, and all of them finished their five daily visits. Descriptive data on 24-hour IOP, acrophase (time of the highest IOP value in a 24-hour cycle), bathyphase (time of the lowest IOP value in a 24-hour cycle), SBP, DBP, and MOPP parameters for the five daily visits among 10 healthy young individuals are described in Table 1. To compare and correlate IOP measurements between GAT and a Tono-Pen AVIA tonometer, we measured IOP in a sitting position using the two tonometers. There was no a significant difference between the lOP readings obtained by GAT and the Tono-Pen AVIA tonometer (p = 0.673). Using Altman-Bland’s method, the mean difference between GAT values and Tono-Pen AVIA values was 0.15 ± 1.09 mmHg, and there was good correlation between the two methods (Table 2). As GAT is considered the clinical standard in tonometry, GAT was used for sitting IOP measurements, while Tonopen-AVIA was used for supine IOP measurements.

**Table 1:** **Descriptive data on intraocular pressure (IOP), systolic and diastolic blood pressure (SBP/DBP), and mean ocular perfusion pressure (MOPP) parameters for five daily visits among healthy young individuals (n = 10)**

		IOP (mmHg)		Acro-phase (h)	Bathy-phase (h)	BP (mmHg)		MOPP (mmHg)	
Subjects	Visits	Sitting ^†^	Supine ^‡^			SBP	DBP	Sitting ^†^	Supine ^‡^
Subject #1	1st	12.25 ± 1.98	12.25 ± 0.89	15	6	118.25 ± 4.13	66.88 ± 4.73	43.75 ± 2.94	42.86 ± 2.09
	2nd	12.63 ± 1.60	12.75 ± 1.04	15	12	121.00 ± 3,89	68.38 ± 2.45	44.65 ± 1.72	40.56 ± 8.09
	3rd	11.88 ± 1.36	11.50 ± 0.93	0	6	120.88 ± 3.40	69.38 ± 3.38	45.82 ± 1.39	45.00 ± 2.16
	4th	12.75 ± 2.05	12.63 ± 2.00	12	6	122.50 ± 3.34	66.88 ± 3.09	44.19 ± 2.24	43.13 ± 2.02
	5th	11.75 ± 1.28	12.75 ± 1.04	21	12	121.38 ± 4.63	68.25 ± 3.62	45.56 ± 2.25	43.28 ± 1.73
Subject #2	1st	13.88 ± 1.25	13.75 ± 1.67	6	12	115.50 ± 5.37	71.63 ± 6.02	43.63 ± 3.48	42.81 ± 3.71
	2nd	13.88 ± 1.36	14.00 ± 1.93	18	3	117.38 ± 3.81	66.75 ± 5.01	41.88 ± 3.22	40.97 ± 3.16
	3rd	14.00 ± 2.14	13.63 ± 1.30	15	3	119.25 ± 3.88	70.38 ± 5.26	43.78 ± 2.51	43.40 ± 3.19
	4th	13.88 ± 0.83	13.88 ± 1.13	12	3	117.13 ± 2.75	66.13 ± 2.42	41.54 ± 1.61	40.85 ± 2.56
	5th	12.88 ± 1.25	13.00 ± 1.41	18	3	117.50 ± 3.89	66.25 ± 4.10	42.68 ± 2.03	41.92 ± 2.59
Subject #3	1st	11.88 ± 0.99	12.88 ± 1.73	12	0	129.13 ± 1.73	72.88 ± 3.68	49.21 ± 1.83	48.15 ± 2.56
	2nd	12.38 ± 1.85	13.75 ± 1.39	3	15	126.25 ± 3.28	74.75 ± 2.05	48.90 ± 1.13	47.64 ± 2.12
	3rd	11.50 ± 1.31	12.88 ± 0.83	21	6	125.50 ± 1.85	70.00 ± 2.98	47.50 ± 1.11	46.01 ± 1.39
	4th	12.13 ± 1.96	13.63 ± 2.00	12	0	126.50 ± 2.62	73.63 ± 2.33	48.71 ± 1.91	46.93 ± 2.92
	5th	12.00 ± 1.20	13.25 ± 1.28	21	0	123.38 ± 2.07	66.00 ± 2.56	44.75 ± 1.45	44.81 ± 1.41
Subject #4	1st	13.38 ± 1.41	13.50 ± 1.69	3	9	115.75 ± 5.09	66.13 ± 2.75	41.74 ± 1.19	41.67 ± 2.84
	2nd	12.63 ± 1.30	14.63 ± 0.92	6	18	116.38 ± 2.13	65.13 ± 1.55	42.18 ± 1.76	40.13 ± 1.70
	3rd	11.75 ± 1.28	13.00 ± 1.41	18	3	115.63 ± 3.66	63.00 ± 1.77	41.94 ± 1.40	40.83 ± 2.08
	4th	11.88 ± 1.25	13.25 ± 0.89	12	3	116.38 ± 3.11	64.88 ± 2.53	42.82 ± 1.93	41.08 ± 0.98
	5th	12.00 ± 1.60	13.38 ± 1.19	6	15	117.75 ± 3.24	66.00 ± 3.25	43.50 ± 2.55	42.21 ± 1.63
Subject #5	1st	12.50 ± 2.07	12.63 ± 1.30	15	3	115.50 ± 5.37	71.63 ± 6.02	45.00 ± 3.18	43.93 ± 3.18
	2nd	13.00 ± 1.77	13.25 ± 1.28	9	0	117.25 ± 2.82	71.75 ± 4.65	44.94 ± 3.10	42.86 ± 4.06
	3rd	13.63 ± 2.20	13.50 ± 1.51	15	3	117.25 ± 4.46	70.88 ± 4.42	43.93 ± 1.51	43.00 ± 1.84
	4th	13.25 ± 1.75	13.38 ± 1.06	18	9	118.25 ± 2.92	71.88 ± 4.58	44.97 ± 2.23	44.71 ± 2.80
	5th	13.50 ± 1.85	13.75 ± 1.58	9	3	118.00 ± 2.88	71.13 ± 3.27	44.33 ± 2.38	44.19 ± 2.08
Subject #6	1st	16.00 ± 1.20	16.63 ± 1.51	6	3	126.88 ± 2.53	73.75 ± 2.38	44.97 ± 1.20	43.54 ± 1.18
	2nd	14.13 ± 1.46	14.50 ± 1.60	18	6	125.63 ± 2.50	70.00 ± 1.85	44.90 ± 1.70	44.11 ± 1.85
	3rd	15.88 ± 1.36	16.75 ± 1.28	6	21	127.75 ± 4.26	72.63 ± 3.38	44.74 ± 1.26	43.36 ± 1.01
	4th	13.00 ± 1.31	13.88 ± 1.64	21	6	126.88 ± 2.36	65.25 ± 1.91	44.19 ± 2.03	43.15 ± 1.59
	5th	13.63 ± 2.62	14.75 ± 1.91	18	6	126.50 ± 1.85	66.00 ± 2.27	43.82 ± 3.31	42.17 ± 2.82
Subject #7	1st	14.50 ± 1.41	14.13 ± 0.99	15	3	119.00 ± 2.45	68.88 ± 4.76	42.56 ± 2.15	43.29 ± 0.96
	2nd	13.88 ± 0.99	14.38 ± 1.41	18	15	117.13 ± 3.36	66.13 ± 2.42	41.54 ± 1.96	40.76 ± 2.71
	3rd	14.88 ± 1.25	14.50 ± 1.07	6	12	116.25 ± 3.45	69.13 ± 4.05	41.68 ± 2.22	41.64 ± 2.64
	4th	13.63 ± 0.92	14.13 ± 1.46	9	3	117.38 ± 2.07	66.25 ± 3.69	41.90 ± 1.75	41.01 ± 2.29
	5th	14.88 ± 1.36	14.63 ± 1.51	18	3	118.25 ± 3.77	66.88 ± 4.73	41.13 ± 3.16	40.82 ± 3.12
Subject #8	1st	11.38 ± 2.07	12.38 ± 1.77	15	3	132.50 ± 5.13	75.13 ± 6.71	51.46 ± 3.52	49.57 ± 2.39
	2nd	12.00 ± 2.00	12.88 ± 2.30	21	0	129.63 ± 2.77	71.00 ± 2.83	48.36 ± 1.48	47.38 ± 1.03
	3rd	12.25 ± 2.19	14.00 ± 2.14	12	3	126.25 ± 2.76	74.50 ± 1.93	48.92 ± 2.14	46.53 ± 3.10
	4th	12.00 ± 0.93	13.25 ± 1.75	18	3	129.75 ± 2.55	69.00 ± 2.33	47.50 ± 1.41	46.50 ± 1.46
	5th	11.38 ± 1.77	12.75 ± 1.28	15	6	128.75 ± 2.43	71.00 ± 2.62	48.79 ± 1.50	47.33 ± 2.35
Subject #9	1st	8.75 ± 0.71	10.25 ± 0.89	12	3	111.13 ± 3.65	67.50 ± 2.07	43.72 ± 8.01	43.22 ± 2.03
	2nd	7.88 ± 1.89	9.50 ± 1.69	15	3	112.38 ± 2.26	65.38 ± 1.77	46.15 ± 1.56	44.06 ± 2.39
	3rd	9.25 ± 1.04	10.13 ± 1.73	3	15	114.00 ± 5.32	67.38 ± 1.92	46.03 ± 1.76	44.15 ± 2.43
	4th	8.75 ± 2.12	10.00 ± 0.93	18	9	116.38 ± 3.50	67.13 ± 2.10	46.94 ± 1.73	44.11 ± 1.60
	5th	8.63 ± 1.85	9.88 ± 0.99	18	15	116.63 ± 2.72	66.38 ± 1.69	46.79 ± 1.32	45.35 ± 0.83
Subject #10	1st	11.75 ± 0.89	12.88 ± 1.13	18	3	125.63 ± 3.66	63.88 ± 2.70	44.56 ± 1.34	43.43 ± 1.66
	2nd	12.38 ± 1.60	13.13 ± 0.99	6	15	124.63 ± 2.88	65.13 ± 2.23	44.26 ± 2.20	43.10 ± 1.30
	3rd	12.88 ± 1.55	13.75 ± 1.16	9	21	125.38 ± 4.07	66.13 ± 2.80	44.60 ± 1.34	42.86 ± 1.46
	4th	12.25 ± 1.04	13.88 ± 1.13	9	18	122.59 ± 1.96	65.13 ± 1.55	44.50 ± 0.89	42.29 ± 1.23
	5th	13.38 ± 1.06	13.50 ± 0.53	9	3	124.13 ± 3.12	64.88 ± 2.53	43.26 ± 1.09	42.50 ± 0.86

^†^Measured by Goldmann applanation tonometry.

^‡^Measured by a Tono-Pen AVIA tonometer (Reichert Technologies Inc., Depew, NY, USA).

Data are expressed as the mean ± standard deviation.

**Table 2:** **Comparison of intraocular pressure (IOP) measured by goldmann applanation tonometry (GAT) and a tono-pen AVIA tonometer (Reichert technologies Inc., Depew, NY, USA) in a sitting position**

	Mean ± SD	Range	p-value
IOP_GAT_ (mmHg)	12.81 ± 2.11	6 ~ 18	0.673*
IOP_AVIA_ (mmHg)	12.92 ± 1.71	6 ~ 19	
Correlation			
Pearson’s *r*	0.788		< 0.001
Linear regression *r*	0.752		< 0.001
Difference^†^ (mmHg)	0.15 ± 1.09	-2 ~ 2	
Linear regression *r* ^‡^	0.052		0.644

*SD* Standard deviation.

*Calculated by student's paired t-test.

^†^IOP_GAT_ minus IOP_AVIA_.

^‡^Calculated by Altman-Bland's method.

The ICCs of the 24-hour IOP measurements are summarized in Table 3. Both in the sitting and supine positions, the maximum and minimum IOPs showed excellent agreement; all ICC values were over 0.900. However, in both positions, the IOP fluctuations showed worse agreement; the ICC value of the sitting IOP fluctuation was just 0.212.

**Table 3:** **Intraclass correlation coefficients (ICC) for comparison of intraocular pressure (IOP) parameters for five daily visits among healthy young individuals (n = 10)**

	Mean ± SD (range)	ICC (95% CI)
Sitting IOP (mmHg)^†^		
Maximum	14.94 ± 1.86 (10 ~ 18)	0.915 (0.792 ~ 0.976)
Minimum	10.52 ± 1.88 (6 ~ 15)	0.933 (0.836 ~ 0.981)
Fluctuation^§^	4.42 ± 1.43 (2 ~ 8)	0.212 (-0.997 ~ 0.779)
Supine IOP (mmHg)^‡^		
Maximum	15.28 ± 1.73 (11 ~ 19)	0.948 (0.870 ~ 0.985)
Minimum	11.40 ± 1.58 (6 ~ 15)	0.917 (0.791 ~ 0.976)
Fluctuation^§^	3.88 ± 1.26 (1 ~ 6)	0.575 (-0.102 ~ 0.882)

*CI* Confidence interval, *SD* Standard deviation.

^†^Measured by Goldmann applanation tonometry.

^‡^Measured by a Tono-Pen AVIA tonometer (Reichert Technologies Inc., Depew, NY, USA).

^§^Difference between the maximum and minimum IOP values observed in a single day.

The ICCs of the 24-hour SBP/DBP measurements for the five daily visits are summarized in Table 4. Similar to the IOP, the maximum and minimum SBP/DBP values showed excellent agreement, regardless of the position; all the ICC values were over 0.800. However, contrary to the IOP results, the SBP/DBP fluctuations showed good to excellent agreement for all visits; most of the ICC values were around 0.800.

**Table 4:** **Intraclass correlation coefficients (ICC) for comparison of systolic and diastolic blood pressure (SBP/DBP) parameters for five daily visits among healthy young individuals (n = 10)**

	Mean ± SD (range)	ICC (95% CI)
Sitting SBP (mmHg)		
Maximum	126.14 ± 5.11 (115 ~ 142)	0.953 (0.884 ~ 0.987)
Minimum	116.64 ± 5.97 (107 ~ 127)	0.971 (0.928 ~ 0.992)
Fluctuation^†^	9.50 ± 2.87 (5 ~ 16)	0.500 (-0.086 ~ 0.847)
Sitting DBP (mmHg)		
Maximum	73.48 ± 4.40 (66 ~ 89)	0.856 (0.643 ~ 0.959)
Minimum	64.20 ± 3.15 (59 ~ 71)	0.859 (0.656 ~ 0.960)
Fluctuation^†^	9.28 ± 3.64 (5 ~ 20)	0.794 (0.502 ~ 0.940)
Supine SBP (mmHg)		
Maximum	127.24 ± 4.46 (118 ~ 135)	0.972 (0.931 ~ 0.992)
Minimum	117.96 ± 6.96 (108 ~ 130)	0.986 (0.965 ~ 0.996)
Fluctuation^†^	9.28 ± 3.59 (3 ~ 18)	0.886 (0.722 ~ 0.967)
Supine DBP (mmHg)		
Maximum	71.74 ± 4.81 (64 ~ 87)	0.906 (0.763 ~ 0.973)
Minimum	62.10 ± 3.44 (51 ~ 70)	0.830 (0.584 ~ 0.951)
Fluctuation^†^	9.64 ± 4.15 (4 ~ 25)	0.837 (0.602 ~ 0.954)

*CI* Confidence interval, *SD* Standard deviation.

^†^Difference between the maximum and minimum BP values observed in a single day.

**Figure 1:**
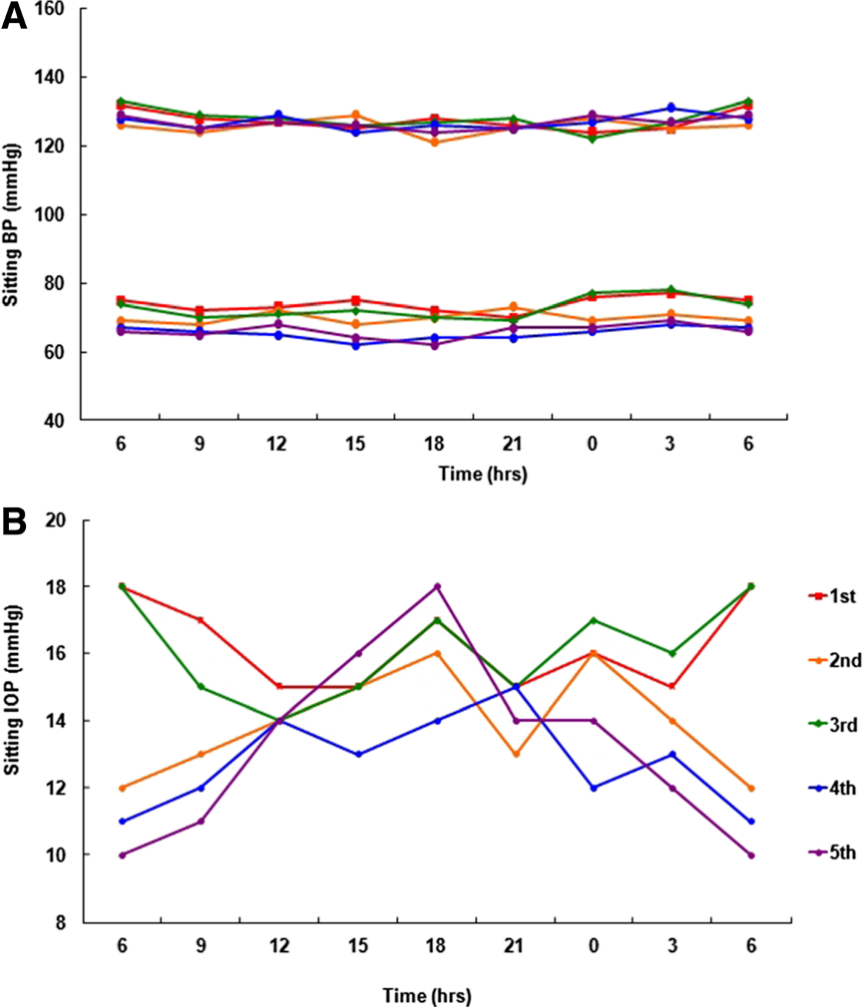
**Representative 24**-**hour circadian profile for the 6th volunteer in the sitting position.** Systolic and diastolic blood pressure **(A)** and intraocular pressure **(B)** were obtained once a week for 5 consecutive weeks. BP = blood pressure, IOP = intraocular pressure.

The ICCs of the 24-hour MOPP parameters are listed in Table 5. In both positions, the maximum and minimum MOPPs showed good to excellent agreement. However, the MOPP fluctuations did not exhibit excellent agreement; the ICC value of the MOPP fluctuation while in the sitting position was the poorest at 0.003. MOPP parameters tended to be similar to IOP parameters.A representative subject who showed unstable 24-hour IOP rhythms is described in Figure 1. Her sitting BPs were very stable for all of her five daily visits (Figure 1A), whereas her sitting IOP pattern differed greatly from day to day (Figure 1B): her sitting IOP results exhibited a concave shape for the first and the third days, while they showed a convex shape for the other three days.

**Table 5:** **Intraclass correlation coefficients (ICC) for comparison of mean ocular perfusion pressure (MOPP) parameters for five daily visits among healthy young individuals (n = 10)**

	Mean ± SD (range)	ICC (95% CI)
Sitting MOPP (mmHg)^†^		
Maximum	47.92 ± 2.65 (44 ~ 59)	0.857 (0.642 ~ 0.959)
Minimum	41.30 ± 4.00 (23 ~ 48)	0.652 (0.146 ~ 0.901)
Fluctuation^§^	6.66 ± 3.67 (3 ~ 26)	0.003 (-1.117 ~ 0.691)
Supine MOPP (mmHg)^‡^		
Maximum	47.04 ± 3.10 (42 ~ 56)	0.931 (0.832 ~ 0.980)
Minimum	39.46 ± 5.10 (21 ~ 48)	0.841 (0.615 ~ 0.954)
Fluctuation^§^	7.48 ± 4.46 (2 ~ 25)	0.670 (0.185 ~ 0.906)

*CI* Confidence interval, *SD* Standard deviation.

^†^Measured by Goldmann applanation tonometry.

^‡^Measured by a Tono-Pen AVIA tonometer (Reichert Technologies Inc., Depew, NY, USA).

^§^Difference between the maximum and minimum MOPP values observed in a single day.

## Discussion

In the present study, we confirmed that 24-hour IOP fluctuations are not highly reproducible on a day to day basis even in healthy young subjects. Regardless of position, the maximum and minimum values of IOP, as well as BP, showed excellent agreement; BP fluctuations also had good to excellent agreement in both positions. However, IOP fluctuations did not show good agreement, especially in the sitting position.

Several studies have evaluated the diurnal and/or circadian IOP rhythms in normal eyes [21–23]; however, only a few reports have investigated the repeatability of IOP rhythms [15, 24]. Liu et al. [22] reported on variations in 24-hour IOP measurements for 91 healthy subjects. They used an automated pneumatonometer rather than GAT, which is most widely accepted for measuring tonometry. Aakre et al. [24] also assessed the reproducibility of IOP measurements in young Caucasians. They also used a noncontact tonometer rather than GAT. Furthermore, they only monitored their subjects for 16 hours and not all 24 hours of a day. In the literature, the first article to report the diurnal IOP patterns using GAT was published by Realini et al. [15]. They investigated diurnal IOP patterns in the eyes of 40 healthy subjects without glaucoma and revealed that diurnal IOP patterns were not repeatable in the short term. Nevertheless, they also did not monitor 24-hour circadian IOP patterns but only 12-hour diurnal IOP patterns in a sitting position from 08:00 A.M. to 08:00 P.M. on two visits one week apart. For each subject, they evaluated the time point-by-time point associations between the two days only. Mottet et al. [25] evaluated the reproducibility of 24-hour IOP rhythms over 6 weeks in six healthy young male subjects; however, they only measured the IOP in a supine position using a pneumatometer, not the gold standard in tonometry. In our study, all subjects underwent IOP assessments over a 24-hour period in sitting and supine positions once a week for 5 consecutive weeks; GAT was used to take measurements in the sitting position and a Tono-Pen AVIA tonometer was used in the supine position.

In the current study, the maximum and minimum IOPs were highly reproducible, while IOP fluctuations were not. Our findings are consistent with that of Realini et al. [11], who reported that nonglaucomatous eyes did not show sustained and repeatable short-term diurnal IOP patterns. However, our ICC values over the 24-hour period showed that IOP fluctuations are much poorer than those found in other reports on both healthy and glaucomatous eyes [21, 22]. This may be because we analyzed data for five daily visits rather than two visits. From a statistical perspective, the more variables involved, the less repeatability. Also, this could be due to the fact that we did not restrain the daily lives of participants during the study; there are many factors that affect IOP in our daily lives. In most previous studies, participants were hospitalized with regular sleep cycles, and their fluid and food intake, including caffeine, as well as their physical activity, were carefully monitored and controlled. Mottet et al. [25] reported that intrasubject homogeneity of distribution over time of the acrophase and bathyphase was significant in three out of six and four out of six subjects, respectively. This is inconsistent with our findings, in which acrophase and bathyphase distributions varied greatly. In their study, however, subjects were housed in a sleep laboratory for 24 hours in a strictly controlled environment (light cycle, temperature, fluid intake, meals) and maintained continuous bed rest with continuous monitoring of sleep at night. The subjects were not allowed to sleep during the day. In our study, we did not restrain our participants’ daily lives in anyway. We wanted to analyze their real IOP rhythms. Thus, the participants underwent IOP and BPs measurements even after exercising and/or drinking a certain amount of caffeine/alcohol, as well as after and/or during working late at night. They also were not restrained from their habitual sleep. If a subject had not slept at night, measurements obtained in the middle of the night (e.g., 3:00 AM) might not reflect their sleeping period rhythm.

Vascular factors are a risk factor for glaucoma development and/or progression. Klein et al. [26] demonstrated that IOP changes are directly and significantly associated with SBP changes. Sehi et al. [27] also reported that DBP significantly influenced IOP over the course of a day in glaucoma patients but not in normal subjects. They hypothesized that glaucoma patients comprised vascular dysfunctions that might have induced the different results between them and normal subjects. In our study, the ICC values of DBP fluctuations had excellent agreement, although the ICC values of IOP fluctuations showed poor agreement. This implies that IOP and BP fluctuations may be positive but not causally correlated. SBP is known to have a circadian rhythm. Reportedly, the BP rise that begins before waking is not associated with physical, but is attributed to a nocturnal decrease in sympathetic activity and circulating catecholamines [12]. However, in our study, the circadian rhythm of BP was not apparent. This might have been affected by irregular sleep patterns, drinking a certain amount of caffeine/alcohol, and working late at night by our subjects.

In our report, IOP values in the supine position were higher than those in the sitting position. These results are consistent with previous evidence that supine pressure measurements are generally higher than those for sitting measurements at the same time point [10, 28, 29].We measured IOPs with GAT in the sitting position and with a handheld Tono-Pen AVIA tonometer in the supine position. These two different tonometers may have different accuracies and respectabilities [30]. However, in our study, not only was there no significant difference between the IOP readings obtained by GAT and Tono-Pen AVIA in the sitting position, but also we recorded good agreement therein. Quaranta et al. [10] also reported that mean daytime Goldmann pressures were not statistically different than nighttime supine Perkins pressures. Although it may induce some measurement errors in a comparative analysis between sitting and supine positions, it may not be an apparent limitation in the investigation of 24-hour IOP fluctuations, because it may affect the absolute values of IOP, but not the rhythm of 24-hour IOP values. Additional studies are required to further investigate circadian IOP rhythms reflecting undisturbed habitual-positional IOP changes with the same tonometer in the sitting and supine positions.

Our study has some limitations that have to be considered. We only included healthy young female subjects. Hence, the current findings cannot be directly extrapolated to male, older, or glaucoma patients. However, considering the influence of age and gender on IOP, we find our study to be well controlled, as the main parameter of this study was ICC, which was calculated as the ratio of the between-subject component of variance to the total variance. Although the study sample size was small, the purpose of this study was to evaluate reproducibility of IOP fluctuations. Thus, the number of times each person was evaluated was more important than the number of subjects that were evaluated. Additionally, central corneal thickness was not considered in this study. Although some studies reported that the 24-hour changes in corneal viscoelasticity do not seem to account for IOP rhythms [12, 31], corneal biomechanical properties may actually influence 24-hour IOP rhythms. Also, our data did not show continuous 24-hour IOP changes, as we only measured IOP and BP every 3 hours over a 24-hour period. If these parameters had been obtained more frequently, the maximum and minimum parameters might have been more accurate. However, this could potentially characterize nonphysiological 24-hour IOP patterns.

## Conclusion

Our study confirmed that 24-hour IOP fluctuations are not highly reproducible and that IOP patterns are not sustained from day to day in healthy young volunteers. Our results imply that a single 24-hour IOP assessment may be not sufficient to characterize circadian IOP patterns for individual subjects.

## Abbreviations

BP: Blood pressure
DBP: Diastolic blood pressure
GAT: Goldmann applanation tonometry
ICC: Intraclass correlation coefficient
IOP: Intraocular pressure
MOPP: Mean ocular perfusion pressure
SBP: Systolic blood pressure
SPSS: Statistical package for social Sciences.
